# Impact of Mantle Cell Lymphoma Contamination of Autologous Stem Cell Grafts on Outcome after High-Dose Chemotherapy

**DOI:** 10.3390/cancers13112558

**Published:** 2021-05-23

**Authors:** Malte Roerden, Stefan Wirths, Martin Sökler, Wolfgang A. Bethge, Wichard Vogel, Juliane S. Walz

**Affiliations:** 1 Department of Hematology, Oncology, Clinical Immunology and Rheumatology, University Hospital Tübingen, 72076 Tübingen, Germany; stefan.wirths@med.uni-tuebingen.de (S.W.); martin.soekler@spitalstsag.ch (M.S.); wolfgang.bethge@med.uni-tuebingen.de (W.A.B.); wichard.vogel@med.uni-tuebingen.de (W.V.); juliane.walz@med.uni-tuebingen.de (J.S.W.); 2 Institute for Cell Biology, Department of Immunology, University of Tübingen, 72076 Tübingen, Germany; 3 Cluster of Excellence iFIT (EXC2180) “Image-Guided and Functionally Instructed Tumor Therapies”, University of Tübingen, 72076 Tübingen, Germany; 4 Clinical Collaboration Unit Translational Immunology, German Cancer Consortium (DKTK), Department of Internal Medicine, University Hospital Tübingen, 72076 Tübingen, Germany; 5 Dr. Margarete Fischer-Bosch Institute of Clinical Pharmacology (IKP) and Robert Bosch Center for Tumor Diseases (RBCT), Auerbachstr. 112, 70376 Stuttgart, Germany

**Keywords:** mantle cell lymphoma, MCL, measurable residual disease, MRD, autologous hematopoietic stem cell transplantation, autologous stem cell graft

## Abstract

**Simple Summary:**

High-dose chemotherapy followed by autologous hematopoietic stem cell transplantation (Auto-HSCT) is a standard frontline treatment for fit mantle cell lymphoma (MCL) patients. As there is a need for predictive factors to identify patients unlikely to benefit from this therapy, we investigated the prognostic impact of lymphoma cell contamination of autologous stem cell grafts. Analyzing a cohort of 36 MCL patients, we show that lymphoma cell contamination of stem cell grafts is associated with poor outcomes after Auto-HSCT. Its analysis might thus improve risk assessment and enable risk-stratified treatment strategies for MCL patients.

**Abstract:**

Novel predictive factors are needed to identify mantle cell lymphoma (MCL) patients at increased risk for relapse after high-dose chemotherapy and autologous hematopoietic stem cell transplantation (HDCT/Auto-HSCT). Although bone marrow and peripheral blood involvement is commonly observed in MCL and lymphoma cell contamination of autologous stem cell grafts might facilitate relapse after Auto-HSCT, prevalence and prognostic significance of residual MCL cells in autologous grafts are unknown. We therefore performed a multiparameter flow cytometry (MFC)-based measurable residual disease (MRD) assessment in autologous stem cell grafts and analyzed its association with clinical outcome in an unselected retrospective cohort of 36 MCL patients. MRD was detectable in four (11%) autologous grafts, with MRD levels ranging from 0.002% to 0.2%. Positive graft-MRD was associated with a significantly shorter progression-free and overall survival when compared to graft-MRD negative patients (median 9 vs. 56 months and 25 vs. 132 months, respectively) and predicted early relapse after Auto-HSCT (median time to relapse 9 vs. 44 months). As a predictor of outcome after HDCT/Auto-HSCT, MFC-based assessment of graft-MRD might improve risk stratification and support clinical decision making for risk-oriented treatment strategies in MCL.

## 1. Introduction

High-dose chemotherapy (HDCT) followed by autologous hematopoietic stem cell transplantation (Auto-HSCT) is standard of care in the front-line management of transplant-eligible mantle cell lymphoma (MCL) patients achieving remission to induction chemoimmunotherapy [1,2]. Early treatment intensification with HDCT/Auto-HSCT improves outcome and enables durable remission in many patients, however, a majority of MCL patients ultimately relapses, then facing a poor prognosis [2,3]. As lymphoma contamination of autologous stem cell grafts might facilitate relapse after HDCT/Auto-HSCT [4,5,6,7], stem cell apheresis is usually performed at the end of induction chemoimmunotherapy. However, although bone marrow (BM) and peripheral blood (PB) involvement is commonly observed in MCL, assessment of measurable residual disease (MRD) in autologous stem cell grafts is not routinely performed and data on the prevalence and prognostic significance of graft-MRD in MCL are scarce [4,5,8,9]. Previous studies on lymphoma cell contamination of autologous grafts in B-cell non-Hodgkin lymphomas (B-NHL) and multiple myeloma were inconclusive with regard to its prognostic significance and often limited by cohort heterogeneity [4,5,6,7,8,9,10,11,12,13].

More recent studies in MCL on the other hand demonstrated that polymerase chain reaction (PCR)-based MRD assessment in PB and BM samples after induction chemoimmunotherapy predicts outcome after Auto-HSCT [14,15,16,17]. However, in part due to the necessity of patient-individual assays, PCR-based MRD assessment is not routinely performed outside clinical trials [18,19,20]. Thus, only the mantle cell lymphoma international prognostic index (MIPI) [21,22,23,24] and post-induction response assessment with computed tomography (CT) are routinely available to identify patients at increased risk for relapse. This is critical considering the growing importance of risk stratification in MCL as (i) the role of maintenance therapy after Auto-HSCT, despite its recommendation by various guidelines [1,25,26], in clinical practice remains unsettled as its benefit is likely to depend on the treatment regimen used for induction and rituximab maintenance is yet to receive formal approval in many countries [1,27,28,29,30,31,32] and (ii) novel therapeutic options have improved the prognosis of MCL patients relapsing after Auto-HSCT in recent years [33,34,35,36,37,38,39,40,41] and might even challenge the status of HDCT/Auto-HSCT in the future.

As multiple studies have demonstrated that response to induction chemoimmunotherapy is of prognostic significance in MCL patients [2,3,14,15,16,17,42,43,44,45], we questioned whether MRD assessment in autologous stem cell grafts predicts outcome after Auto-HSCT. As the characteristic immunophenotype of MCL enables MRD assessment by multiparameter flow cytometry (MFC) [46], we performed an MFC-based analysis of autologous stem cell graft MRD (graft-MRD) and analyzed its association with outcome in an unselected retrospective cohort of 36 MCL patients.

## 2. Materials and Methods

### 2.1. Patients and Samples

A total of 36 patients with histologically confirmed MCL were included in this retrospective analysis based on previous treatment with high-dose chemotherapy and autologous hematopoietic stem cell transplantation (HDCT/Auto-HSCT) and availability of autologous stem cell graft samples for MFC-based MRD assessment. Complete patient characteristics are shown in Table 1. Histological diagnosis was made by an expert pathologist. Patients were treated with HDCT/Auto-HSCT at the University Hospital Tübingen between 2007 and 2020 either as first-line consolidation therapy (*n* = 32) or at relapse (*n* = 4). Response assessment was performed by CT or positron emission tomography (PET)-CT at the end of induction or salvage therapy prior to HDCT/Auto-HSCT based on respective current International Working Group (IWG) criteria [19,47,48].

Apheresis of peripheral blood progenitor cells (PBPC) was performed at the leukapheresis unit of the University Hospital Tübingen, Germany between 2006 and 2020. PBPCs were collected in an outpatient setting with CS3000 PLUS (Baxter, Munich, Germany) or Spectra Optia (Terumo BCT, Lakewood, CO, USA) apheresis systems at the end of induction chemoimmunotherapy. PBPCs were mobilized with chemotherapy followed by daily subcutaneous injection of granulocyte colony stimulating factor (G-CSF, 5 µg/kg body weight). PBPCs were cryopreserved using computer-controlled freezers (IceCube, SY-LAB, Neupurkersdorf, Austria) and Dimethyl sulfoxide (DMSO) containing medium and stored at −196 °C until further use. Separate aliquots stored for quality control were used for graft-MRD assessment.

Informed consent was obtained in accordance with the Declaration of Helsinki protocol. The study was approved by and performed according to the guidelines of the local ethics committee (671/2020BO2).

### 2.2. Multiparameter Flow Cytometry Based MRD Assessment

Thawed cryopreserved PBPCs were stained with an eight-color flow cytometry panel using Zombie Aqua live/dead (BioLegend, Cat# 423101) and the titrated antibodies BV711 anti-human CD19 (Biolegend, Cat# 302245, RRID: AB_2562062), PE anti-human CD22 (Biolegend, Cat# 302506, RRID: AB_2074593), PE/Dazzle anti-human CD5 (Biolegend, Cat# 364011, RRID: AB_2565281), PE/Cy7 anti-human CD23 (Biolegend, Cat# 338516, RRID: AB_2278308), AF-700 anti-human CD45 (Biolegend, Cat# 368513, RRID: AB_2566373), Pacific Blue anti-human CD3 (Biolegend, Cat# 300329, RRID: AB_10552893), Pacific Blue anti-human CD14 (Biolegend, Cat# 325615, RRID: AB_830688), Pacific Blue anti-human CD56 (Biolegend, Cat# 318325, RRID: AB_10612566), Pacific Blue anti-human CD34 (Biolegend, Cat# 343511, RRID: AB_1877198), APC anti-human kappa light chain (Biolegend, Cat# 316509, RRID: AB_493614) and FITC anti-human lambda light chain (Biolegend, Cat# 316606, RRID: AB_493625). All samples were analyzed on a LSR Fortessa flow cytometer (BD Biosciences, Franklin Lakes, NJ, USA).

To define the MFC panel’s sensitivity for MRD detection, serial dilution experiments were performed as previously described [46,49,50,51]. To this end, a control MCL sample (peripheral blood mononuclear cells, PBMCs) was serially 1:10 diluted in healthy volunteer PBMCs or graft-MRD negative PBPCs to assess sensitivity in the context with or without polyclonal B cell background.

For assessment of graft-MRD, a minimum of 10^6^ non-gated events and 500,000 CD45^+^ cells were analyzed [52]. MCL cells were defined as single, viable, lineage (CD3/CD14/CD34/CD56)^−^CD5^+^CD19^+^CD22^+^CD23^−/low^ cells. In accordance with previous studies in MCL and other hematological malignancies [46,53,54,55], positive MRD was defined as a homogeneous cluster of ≥20 cells with the immunophenotypic characteristics defined at diagnosis. Graft-MRD was quantified as % MCL cells of total CD45^+^ cells acquired [52].

### 2.3. Software and Statistical Analysis

Flow cytometric data was analyzed using FlowJo 10.0.8 (BD Biosciences). Graphs were plotted using GraphPad Prism 9 (GraphPad Software, San Diego, CA, USA) and R 4.0.2 (R Foundation for Statistical Computing, Vienna, Austria). Survival curves were computed using the Kaplan-Meier estimate and groups were compared using the log-rank test. Cumulative relapse incidences were computed with R and treating non-relapse mortality as a competing risk, enabling an unbiased analysis [56]. Gray’s test was used to compare cumulative incidence curves between groups (Gray’s test *p* < 0.05 is considered statistically significant) [56,57,58]. Statistical analyses were performed using GraphPad Prism 9, R and SPSS 26 (IBM, Armonk, NY, USA) software. *p* values of < 0.05 were considered statistically significant.

## 3. Results

### 3.1. Patient Characteristics

In total, 36 MCL patients with samples available for graft-MRD assessment were included in this retrospective analysis (Table 1). A total of 29/36 (81%) of patients were male, the median age at diagnosis was 58 years (range 43–69). The median follow-up time after HDCT/Auto-HSCT was 104 months, with only two patients having a follow-up time of less than 12 months. A total of 32/36 (89%) of patients underwent HDCT/Auto-HSCT as consolidating first-line therapy, four (11%) patients were treated with HDCT/Auto-HSCT in a second-line setting.

At diagnosis, most patients (94%) had advanced stage disease (Ann Arbor stage III or IV) and BM involvement was detectable in 28/36 (78%) of patients. In BM histology, MCL infiltration ranged from 1% to 85% of nucleated cells (median 15%). Alternating R-CHOP/R-DHAP was the most frequently used regimen for induction chemoimmunotherapy. Respectively, 22 (61%) and 14 (39%) patients showed a complete (CR) and partial response (PR) at the end of induction therapy prior to HDCT/Auto-HSCT. BEAM (carmustine, etoposide, cytarabine and melphalan [59]) was the standard HDCT regimen and was administered in 27/36 (75%) of patients. TAM (total body irradiation, cytarabine, melphalan [60]) was the second most frequently used HDCT regimen (17%). At Auto-HSCT, a median of 5.2 × 10^6^ (range 2–11 × 10^6^) CD34^+^ cells/kg body weight was re-transfused as autologous stem cell graft.

### 3.2. Outcome after HDCT/Auto-HSCT

**Figure 1:**
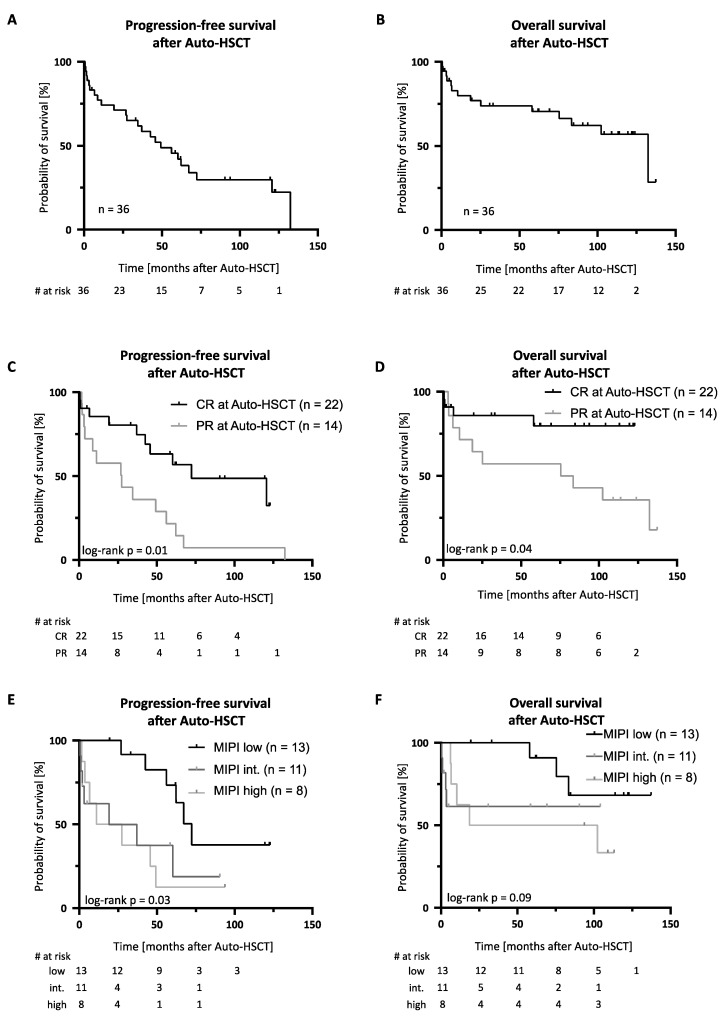
Outcome after high-dose chemotherapy and autologous hematopoietic stem cell transplantation. Kaplan-Meier estimates of (**A**) progression-free (PFS) and (**B**) overall survival (OS) after Auto-HSCT in MCL patient cohort (*n* = 36). (**C**–**F**) PFS and OS in subgroups stratified by (**C**,**D**) remission status prior to Auto-HSCT and (**E**,**F**) MIPI risk group. Abbreviations: Auto-HSCT indicates autologous hematopoietic stem cell transplantation; CR, complete remission, int., intermediate; MIPI, mantle cell international prognostic index; PR, partial remission.

**Figure 2:**
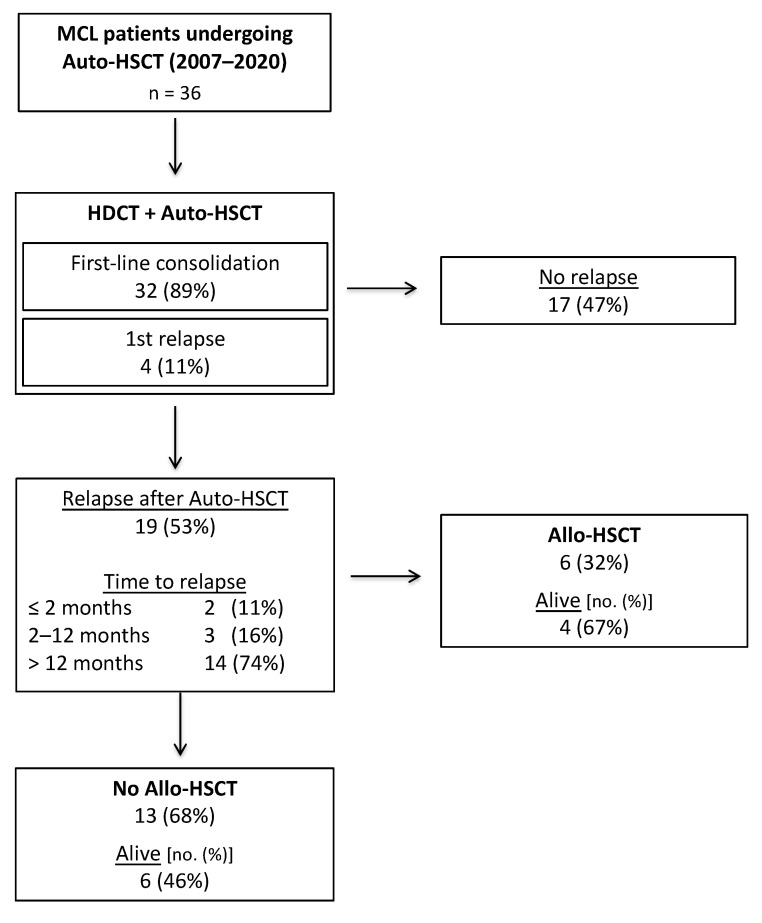
Flow chart of outcome after autologous hematopoietic stem cell transplantation. Outcome and subsequent treatment after Auto-HSCT in MCL patient cohort (*n* = 36). Of 19 patients experiencing relapse after Auto-HSCT, six (32%) later underwent allogeneic HSCT, while 13 (68%) were treated with palliative regimens and/or best supportive care. Abbreviations: Allo/Auto-HSCT indicates allogeneic/autologous hematopoietic stem cell transplantation; HDCT, high-dose chemotherapy; MCL, mantel cell lymphoma; no., number.

**Figure 3:**
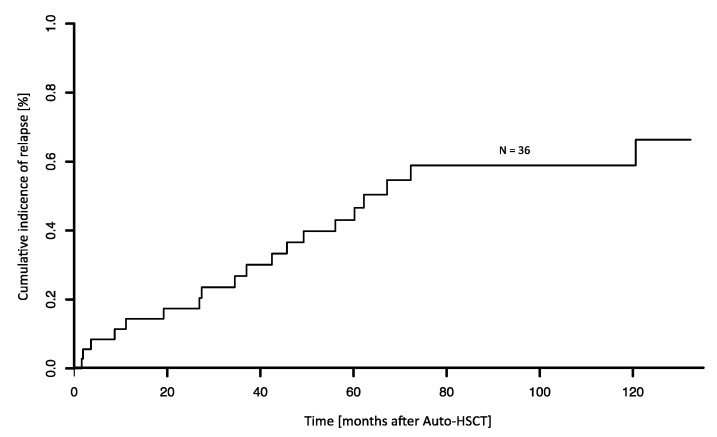
Cumulative incidence of relapse after Auto-HSCT. Cumulative incidence of mantle cell lymphoma relapse after autologous hematopoietic stem cell transplantation (*n* = 36).

After a median follow-up time of 104 months, the 5-year progression free (PFS) and overall survival (OS) after Auto-HSCT were 45.6% (median 49 months) and 70.5% (median 132 months), respectively (Figure 1A,B). Transplant-related mortality (TRM) in HDCT/Auto-HSCT was 8% (3/36 patients). TRM was attributed to infectious complications in two cases. One patient showed autologous stem cell graft failure in the context of Parvovirus B19 infection and died in the course of a subsequent allogeneic HSCT (Allo-HSCT). In total, 19/36 patients (53%) relapsed after HDCT/Auto-HSCT, with most relapses occurring > 12 months after HDCT/Auto-HSCT (Figure 2). Of note, 5/19 (26%) relapsing patients experiencing late disease reoccurrence > 5 years after Auto-HSCT. The cumulative incidence of relapse 5 years after Auto-HSCT was 43% (treating non-relapse mortality as a competing risk, Figure 3).

Achievement of CR prior to Auto-HSCT was associated with a superior PFS and OS when compared to patients in PR after induction chemoimmunotherapy (Figure 1C,D). The 5-year PFS and OS were 63.2% vs. 21.7% and 79.7% vs. 57.1% in patients in CR (*n* = 22) and PR (*n* = 14), respectively. Additionally, risk group allocation according to the MIPI score was predictive of survival after Auto-HSCT in our MCL patient cohort (Figure 1E,F).

Of 19 patients relapsing after Auto-HSCT, 13 (68%) were treated with palliative regimens and showed a 5-year OS after relapse of 29.6% (median 19 months). In total, 6/19 (32%) patients proceeded to Allo-HSCT, achieving a 5-year OS of 60.0% (median not reached). Allo-HSCT related TRM was 12.5% (1/8 patients) in this setting and was attributed to bacterial sepsis.

### 3.3. Sensitivity and Specificity of MFC Panel for MRD Assessment

**Figure 4:**
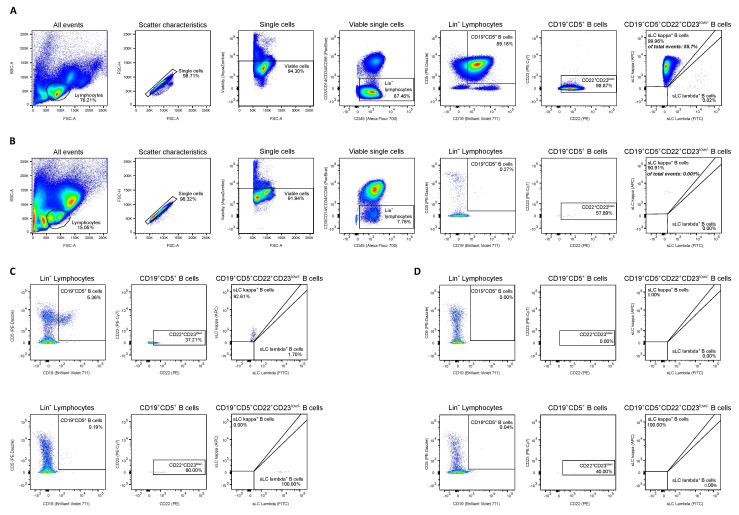
Gating strategy for MFC-based assessment of minimal residual disease in autologous stem cell grafts. MCL cells were defined as single, viable cells negative for the lineage markers CD3/CD14/CD56 and the stem cell marker CD34, low/negative for CD23 and positive for CD5, CD19 and CD22. (**A**) Depiction of gating strategy in a control MCL sample (peripheral blood mononuclear cells). (**B**) The 1:100 000 dilution of control MCL sample in an autologous stem cell graft without measurable residual disease (UPN24). (**C**) Exemplary displays of positive MRD in autologous stem cell grafts of UPN31 (upper panels) and UPN10 (lower panels). (**D**) Exemplary displays of negative MRD in autologous stem cell grafts of UPN7 (upper panels) and UPN16 (lower panels). Abbreviations: FSC indicates forward scatter; lin; lineage; MFC, multiparameter flow cytometry; MRD, measurable residual disease; sLC, surface light chain; SSC, side scatter; UPN, uniform patient number.

Using a homogeneous cluster of ≥20 cells as cut-off for MRD detection and acquiring at least 500,000 CD45^+^ cells (median of acquired CD45^+^ cells 1.5 × 10^6^) gave a theoretical sensitivity of at least 4 × 10^−5^ (0.004%). Facilitated by the lack of polyclonal background after B cell depleting therapy in the patient cohort, serial dilution of a control MCL sample in graft-MRD negative MCL samples showed that lymphoma contamination could be reliably detected with a sensitivity of 10^−5^ (0.001%) MCL cells (Figure 4A,B, Figure S1), which is generally regarded as the limit of detection in MFC-based MRD analyses [20,53,61].

To assess the assay’s applicability for follow-up disease monitoring involving polyclonal B cell background, we evaluated the MFC panel in 10 healthy individuals. Healthy volunteers (HV) showed a median of 0.27% CD19^+^CD5^+^CD22^+^CD23^−/low^ MCL-like cells (Table 2), yielding a limit of detection of 0.64% (LOD, defined as HV mean + 3 standard deviations), unsatisfactory for follow-up MRD analyses. Serial dilution of a control MCL sample in healthy donor PBMCs showed that this matched the actual cut-off for discrimination from polyclonal background, which was between 0.1% and 0.3%.

### 3.4. Frequency and Prognostic Significance of MRD Detection in Autologous Stem Cell Grafts

Of 36 evaluated autologous stem cell graft samples, four (11%) showed graft-MRD above the specified threshold (Figure 4C). MRD levels here ranged from 0.002% to 0.02%, corresponding to estimated absolute numbers of 75 to 1169 re-transfused MCL cells/kg body weight at Auto-HSCT. Of note, MCL cells were not detectable in PB samples of these patients in clinical routine flow cytometry performed after Auto-HSCT (Table S1), suggesting that autologous grafts are more suitable for MRD analysis than PB. In graft-MRD negative patients, the total number of CD19^+^CD5^+^CD22^+^CD23^−/low^ cells ranged from 1 to 10 (mean 1.3, corresponding to 8 × 10^−7^ [0.00008%] MCL-like cells) with 20/32 (63%) of graft-MRD negative samples showing no CD19^+^CD5^+^CD22^+^CD23^−/low^ events (Figure 4D).

**Figure 5:**
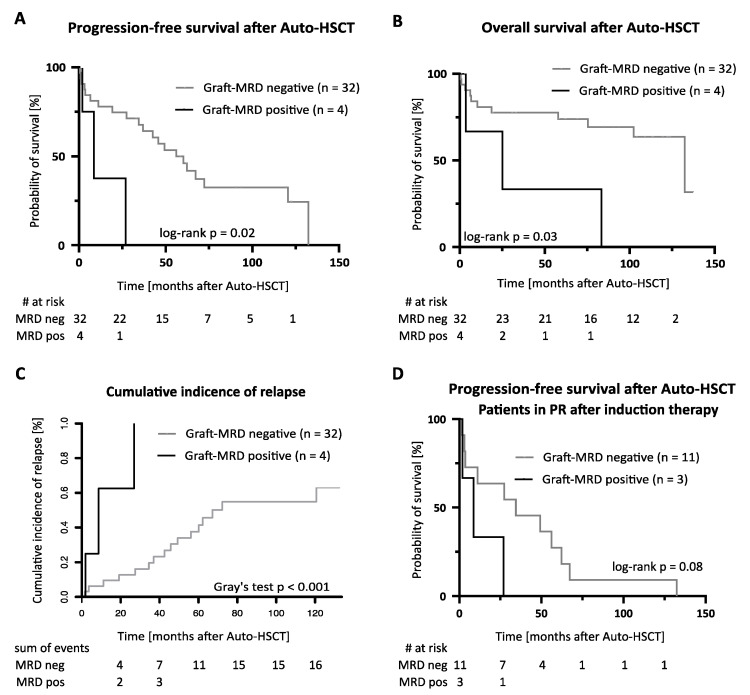
Graft-MRD and outcome after Auto-HSCT. (**A**,**B**) Kaplan-Meier estimates of (**A**) progression-free and (**B**) overall survival in graft-MRD positive and negative patients. (**C**) Cumulative incidence of relapse after Auto-HSCT in graft-MRD positive and negative MCL patients (treating non-relapse mortality as a competing risk). (**D**) Kaplan-Meier estimates of progression-free survival in subgroup of patients in partial remission after induction chemoimmunotherapy. Abbreviations: Auto-HSCT indicates autologous hematopoietic stem cell transplantation; MRD, measurable residual disease; PR, partial remission.

Three graft-MRD positive patients showed a partial remission in CT imaging at the end of induction chemoimmunotherapy, while one patient was in complete remission. No association was observed between graft-MRD and the level of BM infiltration or other disease characteristics at diagnosis (Table 1). Out of four graft-MRD positive patients, three experienced relapse after Auto-HSCT. One graft-MRD positive patient remained in remission at the last follow-up 3 months after Auto-HSCT under ongoing rituximab maintenance therapy. Graft-MRD positive patients showed an inferior PFS and OS (Figure 5A,B) and a higher relapse incidence (Figure 5C) compared to graft-MRD negative patients. The median PFS and OS were 9 vs. 56 and 25 vs. 132 months in graft-MRD positive and negative patients, respectively. The median time to relapse was 9 months in graft-MRD positive patients compared to 44 months in graft-MRD negative relapsing patients (Figure 5D).

In univariable cox regression analyses, graft-MRD was a significant predictor of inferior PFS (HR 4.6 [95% CI 1.2–17.7]) and OS (HR 4.0 [95% CI 1.1–14.9]) after Auto-HSCT (Figure S2). Graft-MRD furthermore predicted a shorter PFS in the subgroup of patients in PR after induction chemoimmunotherapy (*n* = 14, HR 3.6 [95% CI 0.8–16.4], Figure 5D) and when only considering patients undergoing Auto-HSCT as first-line consolidation (Figure S3). In a multivariable cox regression analysis including the variables *MIPI risk group* and *remission status at Auto-HSCT* the hazard ratios of graft-MRD for PFS and OS were 2.5 [95% CI 0.4–14.8] and 2.1 [95% CI 0.3–14.2], respectively (Figure S2).

## 4. Discussion

Identifying MCL patients at increased risk for relapse after HDCT/Auto-HSCT is of growing importance as novel therapeutic options, including bruton tyrosine kinase (BTK) inhibitors, immunomodulatory agents and chimeric antigen receptor (CAR) T cell therapies have improved the prognosis of MCL patients relapsing after HDCT/Auto-HSCT and might even challenge the status of HDCT as standard frontline treatment in the future [33,34,35,36,41]. Furthermore, at-risk patients might benefit particularly from maintenance therapy after Auto-HSCT, the significance of which remains unsettled in the context of different induction therapy regimens [1,30,31,32]. Aiming to improve prediction of outcome after Auto-HSCT, we here present a first analysis of autologous stem cell graft MRD in MCL patients after B cell depleting induction chemoimmunotherapy.

While both MIPI score and response assessment with (PET-) CT [42,43] are well established predictors of outcome after Auto-HSCT, MRD assessment has not yet become clinical routine outside of clinical trials in MCL. For PCR-based analyses, the current gold standard, this is in part due to the necessity to establish patient-individual assays in most patients [14,15,16,17]. In addition to PCR, a recent study demonstrated the capacity of an MFC-based MRD assessment in BM and PB samples to predict outcome in MCL [46]. MFC (using a cut-off of 10^−4^ [0.01%]) here showed true-positive and true-negative rates of 80% and 92% with respect to PCR, respectively. In the study at hand, acquisition of high numbers of events/lymphocytes [61] and the lack of polyclonal background after B cell depleting induction chemoimmunotherapy allowed for an even higher sensitivity in graft-MRD detection of 10^−5^ (0.001%). One downside of the reliance on a lack of polyclonal B cell background, however, is the lacking suitability of our MFC panel for long-term disease monitoring (e.g., in PB or BM samples) after Auto-HSCT. On the other hand, the MFC panel used in our study is based on cell surface markers generally assessed at diagnosis and is thus more widely applicable than MFC panels relying on the detection of markers not necessary assessed in clinical routine flow cytometry to discriminate MCL cells from polyclonal B cell background (e.g., LAIR and CD11a [62]). Additionally, our assay enables MRD assessment even in the absence of diagnostic samples, as only a minority of MCL cases deviate from the typical immunophenotype [63,64,65].

Overall, PFS and OS after Auto-HSCT in our MCL patient cohort were comparable with previous reports of MCL patients undergoing intensive frontline therapy [2,3,8,17,45]. Of note, despite the analysis reaching far back, frontline therapy was very homogenous within the cohort and remains representative for current treatment recommendations [1,25]. The homogenous pretransplant management thereby minimizes confounding variables in the analysis of predictive factors for the outcome after Auto-HSCT. Despite the limited cohort size and the resulting need to interpret the data with caution, we could demonstrate that graft-MRD is associated with an inferior PFS and OS after Auto-HSCT. As particularly the graft-MRD positive group was limited in size, larger and ideally prospective follow-up studies will be necessary to validate the findings of our study and will also allow to delineate potential confounding variables.

Of note, the prevalence of graft-MRD was lower than reported in PCR-based MRD studies using PB and/or BM samples [14,15,46]. In addition to the higher sensitivity of PCR, this might be due to non-mobilized BM-residing lymphoma cells not detectable in PBPC graft analysis. Comparative MRD analyses of stem cell grafts and BM samples will be necessary to define the sensitivity of graft-MRD in detecting residual BM disease.

While also detectable in one patient in CR, graft-MRD was primarily observed in patients not achieving a complete response to induction chemoimmunotherapy. Of note, graft-MRD was associated with a shorter PFS in this subgroup and predicted early relapse after Auto-HSCT. Based on these observations, assessment of graft-MRD might be of particular relevance in patients not achieving a CR prior to Auto-HSCT.

It remains to be elucidated whether autografted lymphoma cells actually contribute to an unfavorable disease course or graft-MRD rather reflects aggressive disease biology and residual in vivo disease as an indicator of a particularly poor response to induction therapy. As such and due to its association with early relapse, detection of graft-MRD raises the question whether HDCT/Auto-HSCT remains the adequate treatment strategy in these poor-responding patients. Both BTK inhibitor-based and CAR T cell therapy, despite a lack of data in this setting, here represents promising and legitimate alternatives [35,36,41]. While future studies need to define the role of BTK inhibitors and CAR T cell therapy in patients not achieving a CR after induction chemoimmunotherapy, positive graft-MRD already advocates for maintenance therapy and close follow-up disease monitoring after Auto-HSCT. Additional, larger studies are necessary to define the significance of graft-MRD in MCL patients achieving a CR after induction chemoimmunotherapy and whether ex vivo graft purging might be of benefit in graft-MRD positive patients [66,67,68].

## 5. Conclusions

We here show for the first time that MRD assessment in autologous stem cell grafts by MFC, a non-invasive, inexpensive and well-established method, predicts outcome after high-dose chemotherapy. As a predictor of early relapse and poor outcome after HDCT/Auto-HSCT, graft-MRD assessment might contribute to pretransplant risk assessment and supports clinical decision making for risk-oriented treatment strategies in MCL patients.

## Figures and Tables

**Table 1:** Patient characteristics.

Characteristic	All Patients	Graft-MRD Negative Patients	Graft-MRD Positive Patients	*p*
**Demographics**				
*Number of patients*	36	32	4	
*Follow-up time (months) ^#^*	104	104	Undefined	
*Male (no.[%)]*	29 (81)	26 (81)	4 (100)	1.0 ^†^
*Median age (range)*	58 (43–69)	58 (43–69)	57 (53–62)	1.0 ^$^
**Disease characteristics** (no.[%)]				
*Stage I–II*	2 (6)	2 (6)	0 (0)	0.70 ^§^
*Stage III–IV*	34 (94)	30 (94)	4 (100)	
*BM involvement at diagnosis*	28 (78)	24 (75)	4 (100)	0.56 ^†^
*Extranodal disease at diagnosis*	14 (39)	12 (38)	2 (50)	0.63 ^†^
**MIPI risk group** (no.[%)]				0.39 ^§^
*Low risk*	13 (36)	12 (38)	1 (33)	
*Intermediate risk*	11 (31)	9 (28)	1 (33)	
*High risk*	8 (22)	8 (25)	1 (33)	
*n.a.*	4 (11)	3 (9)	1 (33)	
**First-line therapy** (no.[%)]				0.08 ^§^
*R-CHOP / R-DHAP*	27 (75)	25 (78)	2 (50)	
*R-CHOP*	4 (11)	4 (13)	0 (0)	
*Other (Rituximab-containing)*	5 (14)	3 (9)	2 (50)	
**Auto-HSCT setting**				0.39 ^†^
*First-line consolidation*	32 (89)	29 (91)	3 (75)	
*Second-line therapy*	4 (11)	3 (9)	1 (25)	
**Response prior to Auto-HSCT**				0.28 ^†^
*CR*	22 (61)	21 (66)	1 (25)	
*PR*	14 (39)	11 (34)	3 (75)	
**Mobilization regimen** (no.[%)]				1.0 ^†^
*R-DHAP*	29 (81)	26 (81)	3 (75)	
*Other*	7 (19)	6 (19)	1 (25)	
**HDCT regimen** (no.[%)]				0.39 ^†^
*BEAM*	27 (75)	23 (72)	4 (100)	
*Other*	9 (25)	9 (28)	0 (0)	
**Maintenance therapy** (no.[%)]				0.76 ^§^
*Rituximab*	5 (14)	4 (13)	1 (25)	
*Ibrutinib*	1 (3)	1 (3)	0 (0)	
*None*	30 (83)	27 (84)	3 (75)	
**Median CD34^+^ cell count / kg body weight** (range)				
*Total harvested*	8.0 (4–29)	7.9 (4–29)	11.3 (9–14)	0.10 ^$^
*Re-transfused at Auto-HSCT*	5.2 (2–11)	5.0 (2–11)	5.8 (4–7)	0.53 ^$^

Characteristics of MCL patient cohort. Variables are displayed in bold, characteristics within each variable in italics (left column). Abbreviations: Auto-HSCT indicates autologous hematopoietic stem cell transplantation; BEAM, carmustine (BCNU)/etoposide/cytarabine/melphalan; HDCT, high-dose chemotherapy; HSCT, hematopoietic stem cell transplantation; MIPI, mantle cell lymphoma international prognostic index; n.a.; not available; no., number; R-CHOP, rituximab/cyclophosphamide/doxorubicin/vincristine/prednisone; R-DHAP, rituximab/dexamethasone/high-dose cytarabine/cisplatin; #, reverse Kaplan-Meier estimate; $, Mann-Whitney-U test; §, Chi-squared test and †, Fisher’s exact test.

**Table 2:** MCL-like cells in healthy volunteers.

Unit	Total Events	CD45 ^+^ Cells	CD19 ^+^ CD5 ^+^	% CD19 ^+^ CD5 ^+^	CD19 ^+^ CD5 ^+^ CD22 ^+^ CD23 ^low/^^−^	% CD19 ^+^ CD5 ^+^ CD22 ^+^ CD23 ^low/^^−^
**Events** Median (SD)	1,027,505 (238,823)	701,150 (215,308)	5382 (4443)	0.64 (0.53)	1650 (750)	0.27% (0.12)
**LC ratio Kappa/Lambda** Median (SD)					1.67 (0.65)	
**Level of blank**					3144	0.48%
**Level of detection**					4160	0.64%

Frequency of B cell subpopulations and MCL-like cells in peripheral blood mononuclear cells of healthy volunteers (n = 10). Percentages of subpopulations are depicted in respect to total CD45^+^ events. Level of blank is defined as healthy volunteer mean + 1.645 SD; Level of detection is defined as healthy volunteer mean + 3 SD. Abbreviations: LC indicates light chain ratio and SD, standard deviation.

## Data Availability

The data supporting the findings of this study are available within the article and its supplementary materials.

## Supplementary Materials

The following are available online at https://www.mdpi.com/article/10.3390/cancers13112558/s1, Table S1: Comparison of graft-MRD with clinical routine assessment of remission status, Figure S1: Serial dilution of control MCL sample, Figure S2: Univariable and multivariable cox regression analysis, Figure S3: Treatment setting and outcome after autologous HSCT.
