# Supplementary material for: Impact of Mantle Cell Lymphoma Contamination of Autologous Stem Cell Grafts on Outcome after High-Dose Chemotherapy
Source: Cancers (Basel). 2021 May 23;13(11):2558. doi: 10.3390/cancers13112558 (PMC8197101; doi:10.3390/cancers13112558)
Supplement: Supplementary file 1 [file cancers-13-02558-s001.zip › cancers-1182035-supplementary.pdf]

cancers

Supplementary Materials

Impact of Mantle Cell Lymphoma Contamination of Autologous Stem Cell Grafts on Outcome After High-Dose Chemotherapy

Malte Roerden, Stefan Wirths, Martin Sökler, Wolfgang A. Bethge, Wichard Vogel and Juliane S. Walz

Graft-MRD	Pre-transplant analysis			Post-transplant analysis	
	PB-FC	BM-FC	BM-PCR	PB-FC	BM-FC
Negative (*n* = 21)<br>% Positive (no. analyzed)	0 (7)	0 (1)	0 (6)	0 (16)	0 (6)
Positive (*n* = 3)<br>% Positive (no. analyzed)	n.d.	n.d.	n.d.	0 (3)	100 (1)

Table S1. Comparison of graft-MRD with clinical routine assessment of remission status. Comparison of graft-MRD analysis with data from clinical routine flow cytometry-based analysis of remission status in the respective last and/or first analysis before and after autologous hematopoietic stem cell transplantation. Data shown for patients for whom data from at least one timepoint were available (n = 24). Abbreviations: BM, bone marrow; FC, flow cytometry; MRD, measurable residual disease; no., number; n.d., not done and PCR, polymerase chain reaction.

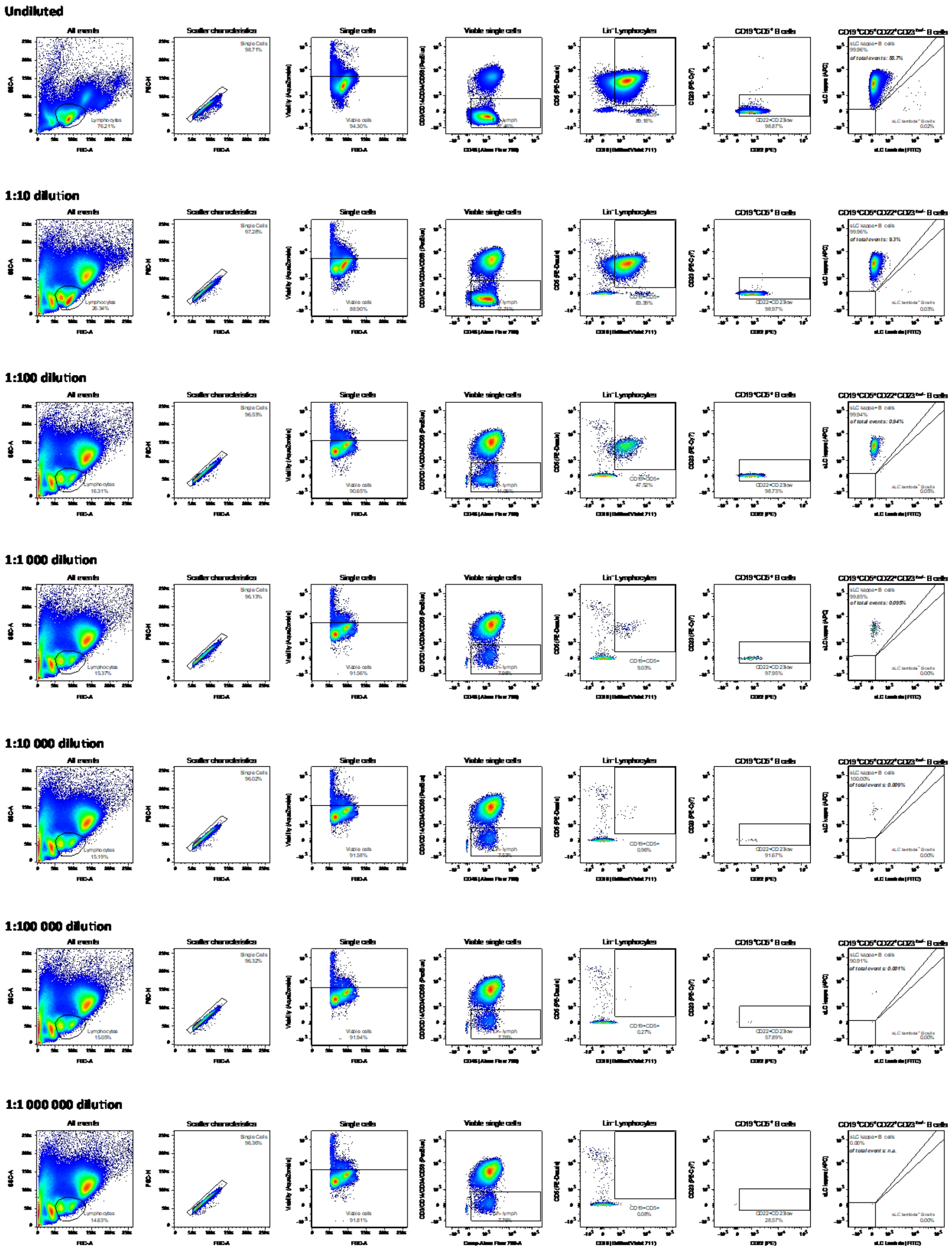

Figure S1: Serial dilution of control MCL sample. Assessment of multiparameter flow cytometry panel sensitivity for detection of measurable residual disease by Serial dilution of a control mantle cell lymphoma sample in an autologous stem cell graft without measurable residual disease.

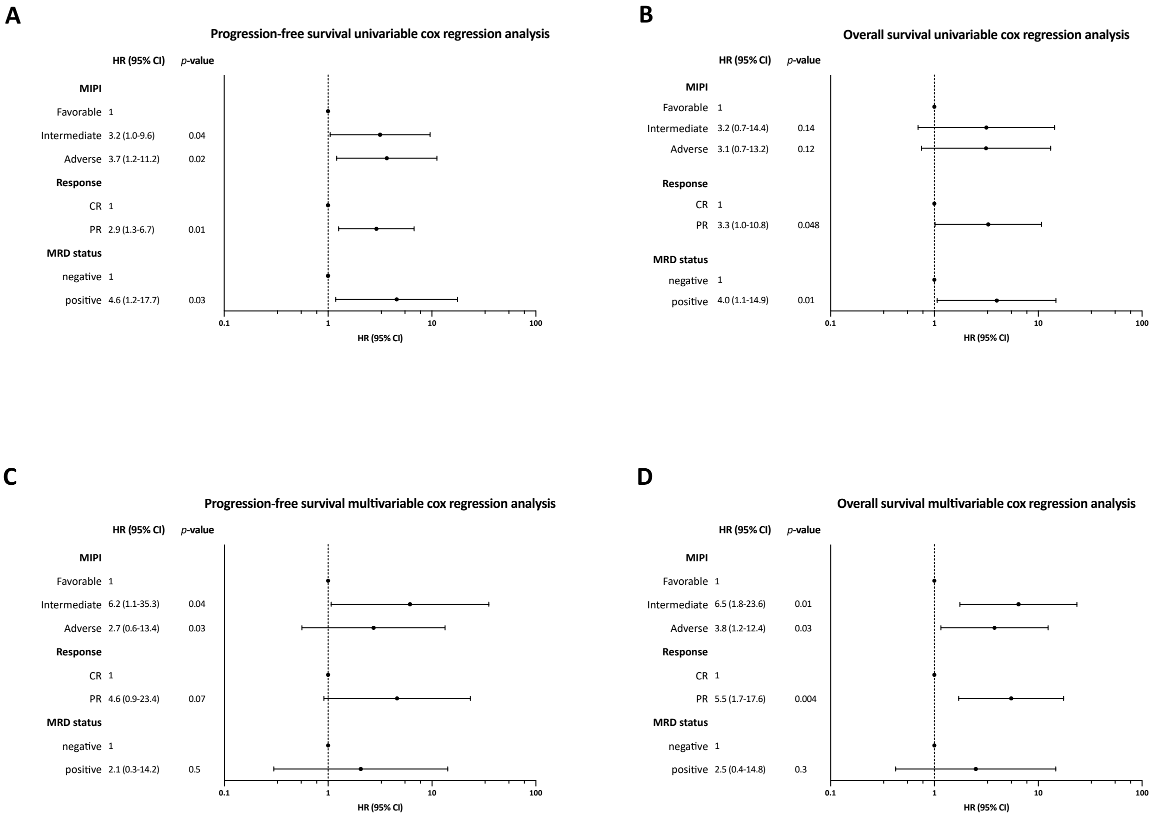

Figure S2: Univariable and multivariable cox regression analysis. Cox regression analysis of determinats for survival after autologous hematopoietic stem cell transplantation.

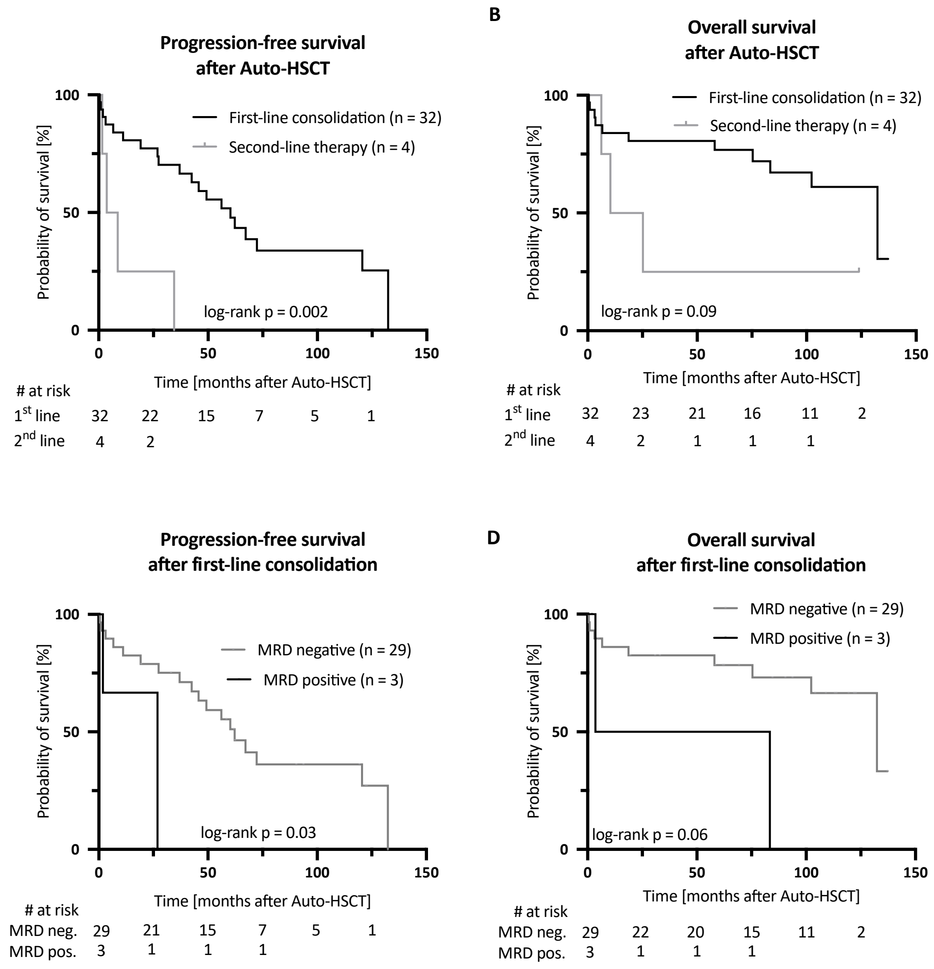

Figure S3: Treatment setting and outcome after autologous HSCT. (**A-B**) Kaplan-Meier estimates of (**A**) progression-free (PFS) and (**B**) overall survival (OS) grouped by treatment setting (first-line consolidation versus second-line therapy). (**C-D**) Kaplan-Meier estimates of (**C**) PFS and (**D**) OS in the subgroup of patients undergoing autologous HSCT as first-line consolidating therapy (n = 32).

© 2021 by the authors. Licensee MDPI, Basel, Switzerland. This article is an open access article distributed under the terms and conditions of the Creative Commons Attribution (CC BY) license (http://creativecommons.org/licenses/by/4.0/).
